# An innovative gamified gait biofeedback interface targeting propulsion: feasibility and preliminary effects

**DOI:** 10.21203/rs.3.rs-8099435/v1

**Published:** 2025-12-09

**Authors:** Bennett L Alterman, Alexandra Slusarenko, Minuk Kim, Zahin Alam, Deborah Fowler, Shilpa Krishnan, Maribeth Gandy Coleman, Steven L Wolf, Trisha M Kesar

**Affiliations:** Emory University School of Medicine; Emory University School of Medicine; Emory University School of Medicine; Emory University School of Medicine; Savannah College of Arts and Design (SCAD); Emory University School of Medicine; Georgia Institute of Technology; Emory University School of Medicine; Emory University School of Medicine

**Keywords:** stroke, biofeedback, gait, gamification, biomechanics, treadmill walking

## Abstract

**Background:**

Enhancing the efficacy of gait rehabilitation is an important area of need as most persons with a history of stroke continue to experience gait deficits following discharge from rehabilitation. Incorporating gamification and real-time biofeedback into gait retraining may provide benefits by increasing engagement and salience of stepping practice to target specific gait parameters, maximizing therapeutic impact on walking function. The objective of this study was to evaluate the feasibility and preliminary effects of a novel, customized, gamified gait biofeedback interface specifically designed to enhance propulsion during gait training.

**Methods:**

A repeated-measures design was used to compare 3 speed-matched treadmill walking bouts: (1) walking without biofeedback (noBF); (2) walking while receiving simple, real-time audiovisual conventional biofeedback (cBF); and (3) walking while receiving gamified, real-time audiovisual biofeedback (gBF). Gait biomechanics, physiological intensity, and self-reports of task workload, user experience, and engagement were obtained from 15 participants (9 able-bodied, 6 post-stroke).

**Results:**

Compared to noBF, both gBF and cBF showed significantly greater increases in peak anterior ground reaction force, trailing limb angle, and ankle moment of the targeted leg during biofeedback exposure (Min. 3) and during the post-test without biofeedback exposure (Post), with moderate to large effect sizes. Compared to walking without biofeedback, both biofeedback conditions induced significantly greater physiological intensity (heart rate and rating of perceived exertion). NASA-Task Load Index results showed that gBF induced a higher workload than cBF for mental demand, temporal demand, performance, frustration, and effort. Also, gBF was rated higher in the novelty component of the User Experience Questionnaire.

**Conclusions:**

This preliminary study confirmed the feasibility of real-time gamified gait biofeedback, suggesting that gBF can induce similar enhancements in gait biomechanics and physiological intensity as cBF, while promoting greater task load and mental demand during walking practice. This work lays foundations for future studies that further refine and customize the game design, as well as evaluate the effects of gBF in larger samples and greater training dosage.

**Trial registration:**

This study was registered on ClinicalTrials.gov (NCT04013971) and all study procedures were approved by the human subjects review board (IRB00106866). The study started 04/04/2022 and ended 03/31/2023.

## Introduction

Stroke is a leading cause of mortality and adult disability worldwide.^1, 2^ Most persons with a history of stroke have residual gait deficits that limit walking speed, endurance, and community participation, even after discharge from rehabilitation.^3–8^ Thus, there remains a need to develop new interventions and to improve existing gait retraining paradigms to provide more robust biomechanical and functional rehabilitation outcomes for persons with a history of stroke.^9^

Post-stroke biomechanical impairments, such as reduced swing phase knee and ankle flexion, impair proper gait function and increase the risk for falls.^4, 7^ Reduced paretic propulsion during terminal stance is another important post-stroke gait deficit associated with reduced gait speed, inter-limb symmetry, swing phase knee flexion, and walking function.^10–14^ Previous research has shown that paretic propulsion can be enhanced with gait retraining, and that training-induced change in propulsion may be a predictor of clinical response to gait training.^10, 15–19^ Thus, reduced paretic propulsion is an important therapeutic target for post-stroke gait retraining.

There are few gait training approaches designed to optimize motor learning of improved gait patterns by targeting specific deficits and focusing practice preferentially on the paretic leg. Recent research trends have tried to modify the dosage or intensity of existing gait rehabilitation, or tested complementary interventions such as neuromodulation.^9, 20, 21^ Robotic and exoskeleton assistive devices also show potential, but are often high-cost and require specialized training and supervision to operate.^22^ Furthermore, extensive stepping practice is required to induce sustained improvements in gait patterns, but the number of repetitions of stepping practice pragmatically feasible in clinical treatment sessions has been remarkably low.^17, 23–27^ To maximize the therapeutic impact of each session of clinical rehabilitation, there is a need to increase participant engagement, motivation, and salience during training to promote greater neuroplasticity and motor learning.^24, 25^ Real-time biofeedback is one gait rehabilitation strategy that may address these needs.

Biofeedback is a method of providing information about ongoing movement performance (e.g., joint angles, muscle activity) or physiological parameters (e.g., heart rate variability) during motor training to induce self-regulated changes in behavior, biomechanics, or activity.^28^ Real-time biofeedback has emerged as a promising post-stroke gait training strategy that enhances an individual’s awareness of the impairment targeted during gait training.^29–31^ Previously, gait biofeedback was used to modulate electromyographic activity and step length asymmetry in people with post-stroke hemiparesis.^32–37^ Similarly, treadmill training combined with unilateral audiovisual real-time gait biofeedback increased propulsion in the targeted/paretic leg in persons with a history of stroke (ST) and able-bodied (AB) individuals, while the non-targeted/non-paretic leg remained unaffected.^30, 38–40^ However, most prior gait biofeedback work was conducted using simple, non-engaging visual interfaces. Thus, the overall goal of the current study was to develop and evaluate the feasibility of a more engaging *game-based* gait biofeedback interface that may promote greater motivation and better user experiences during training.

“Gamification” refers to the use of video game elements in non-gaming systems (such as rehabilitation) to improve user engagement and shape user behavior.^41–43^ Games are designed to be more interesting and enjoyable than traditional therapy or motor training tasks, thereby encouraging greater repetitions of high-quality movement patterns.^44–47^ Games provide interactive, real-time, experiential learning facilitated via computer interfaces, and enable objective measurement and shaping of behavior in challenging but safe environments, while maintaining standardization of treatment characteristics.^48^ Games use cues to provide accurate and immediate feedback about movement, reward desirable motor performance, and discourage maladaptive behavior, while also being engaging and motivating.^47, 49–51^ Games can be personalized to each individual by modifying task-difficulty according to baseline impairments, and designing task-specific programs that are meaningful to participants—considerations that increase motor learning.^44, 45, 50, 51^ For rehabilitation clinicians, game-based therapies provide the advantage of immediate, quantitative feedback about motor performance and enable customization of challenge during therapy.^44, 46, 47, 50, 51^ Furthermore, gamified biofeedback may be advantageous for engaging explicit and/or exploratory learning processes as well as enhancing salience and attentional engagement, promoting greater neuroplasticity.^52, 53^

Technological advancements during the last decade have led to new types of interfaces for recreational gaming, and many off-the-shelf games have been used in rehabilitation.^44–47^ However, intuitive and engaging games designed to target specific gait deficits, and for use specifically in neurorehabilitation, are not currently available.^46, 47^ The objectives of the current study were to: (1) demonstrate the feasibility of a novel game-based biofeedback interface targeting paretic propulsion for gait retraining; and to (2) conduct a preliminary dose-matched comparison of the effects of conventional versus gamified gait biofeedback on gait biomechanics, physiological intensity during training, and user experience and engagement. We hypothesized that compared to conventional gait biofeedback (cBF), gamified gait biofeedback (gBF) would demonstrate similar changes in biomechanics and physiological intensity. We also hypothesized that participants would show greater engagement and motivation, as well as lesser boredom during gBF versus cBF.

## Materials and methods

### Participants

A total of 15 participants (9 able-bodied (age = 27.9 ± 4.1 years, 5 females) and 6 post-stroke (age = 63.0 ± 7.7 years, 2 females)) completed this study (Table 1). All participants provided written informed consent and the Emory University Institutional Review Board approved all methods. Inclusion criteria included ≥ 6 months elapsed since a left-hemisphere cortical or subcortical stroke, ability to walk on a treadmill for ≥ 2 min at a speed ≥ 0.5 m/s, and ability to communicate with the study team. Exclusion criteria included orthopedic or neurological conditions preventing walking, neurologic diagnosis other than stroke, cerebellar signs, and inability to communicate with the study team. Able-bodied individuals were included if they did not have any neurologic or musculoskeletal conditions affecting their ability to complete the study procedures. A total of 19 (10 AB, 9 ST) people were screened and consented for the study; data from 4 participants had to be excluded from the study (2 ST participants did not meet minimum walking speed requirements, 1 ST participant was excluded due to right-hemispheric stroke, 1 AB participant was excluded due to loss of Post timepoint data).

### Setup for 3-D gait biomechanics

Reflective markers were attached to bilateral thighs, shanks, shoes, pelvis, and trunk.^54^ Marker data were collected using a 7-camera motion capture system at a sampling rate of 120 Hz (Vicon Inc., Oxfordshire, UK). A 6-component force platform (Bertec Corporation, Ohio, USA) embedded below each belt of the split-belt treadmill was used to record ground reaction forces during walking bouts.

### Determination of self-selected gait speed

Participants were instructed to walk while the treadmill speed was gradually increased by 0.1 m/s increments until a comfortable walking pace was self-selected. A comfortable walking pace was defined as the pace at which the participant walks during everyday activity at an easy pace. For safety, participants were supported with an overhead harness, and instructed to grasp a handrail for stability. None of the ST participants wore an orthosis during the treadmill walking tests. An emergency shut-off switch could be used at any time by the experimenter to stop the treadmill.

### Experimental design

**Figure 1:**
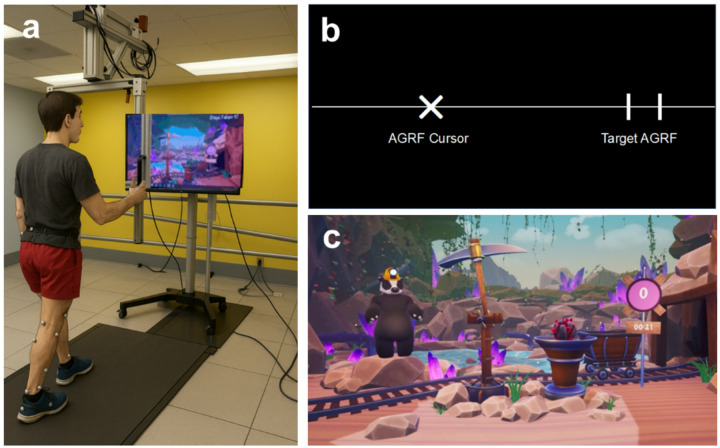
Overview of experimental setup. (a) For the gait biofeedback conditions, participants walked on an instrumented treadmill with a screen and speaker in front of the treadmill that provided audiovisual biofeedback targeting increased peak AGRF generation for the right leg in able-bodied participants (AB) and individuals with right-sided post-stroke hemiparesis (ST). (b) Visualization of the conventional biofeedback interface display (cBF). Vertical lines denote the target AGRF window, and an “X” representing the antero-posterior GRF moves leftward or rightward during the gait cycle. Success is denoted by visual cues (the “X” reaches and/or exceeds the target window and an auditory beep). (c) Visualization of the novel gamified biofeedback display (gBF). A colorful, engaging scene themed around mining and a custom-designed character (badger) displays a pickaxe that moves up and down, representing ongoing antero-posterior GRF values during gait. The target AGRF and current AGRF are displayed as a numeric value near the pickaxe. Success is denoted by visual display (gems being mined) and auditory celebratory musical cues.

Participants completed three 4-min bouts at the same self-selected speed: (1) a control bout (noBF), where participants walked on the treadmill without biofeedback; (2) a conventional biofeedback (cBF) bout, where participants walked using conventional real-time audiovisual biofeedback; and (3) a gamified biofeedback (gBF) bout, where participants walked while receiving game-based, real-time audiovisual biofeedback (Fig. 1a). Participants were given scripted instructions on the mechanics of each biofeedback interface (i.e., how the game works) and on the training task (i.e., increase propulsion with the paretic/right leg). The noBF bout was completed first, followed by the biofeedback bouts. The order of the biofeedback bouts was semi-randomized between participants; half of the participants completed cBF before gBF, while the other half completed gBF before cBF.

For the noBF bout, participants walked for 4 min at their self-selected speed. For both cBF and gBF, participants also walked for 4 min, with the first 30 s comprising walking without biofeedback (Pre), followed by 3 min walking with biofeedback, and immediately followed by another 30 s period walking without biofeedback (Post), all at the same gait speed. Thus, the participants were exposed to biofeedback for a matched 3 min duration during both cBF and gBF.

### Determination of target anterior ground reaction force (AGRF) for biofeedback

Participants walked at their self-selected speed for 30 s to establish a baseline AGRF, calculated as the average of the five highest AGRF peaks recorded from the paretic (ST) or right (AB) leg via force platforms embedded within the split-belt treadmill. To determine the target AGRF % level, participants completed a short gait trial where biofeedback was set at an initial 20% above baseline AGRF. If the participant demonstrated > 50% success with reaching the target AGRF over 10 gait cycles, the target AGRF was increased by 5% for the subsequent 10 gait cycles, until the participant demonstrated < 50% success rate during biofeedback without excessive handrail support, effort, or compensatory gait patterns (judged by experimenters through visual inspection). Thus, the target AGRF for biofeedback was determined in an individual-specific manner with the goal of optimizing the challenge level during gait biofeedback. The same target AGRF percentage was used during both conventional and gamified biofeedback bouts.

#### Biofeedback methodology

##### Conventional biofeedback (cBF)

A screen and speaker were positioned in front of the participant for relaying visual and audio biofeedback. Similar to prior work, visual biofeedback consisted of a horizontal black line featuring a sliding “X” displaying current real-time AGRF during the stride cycle from the paretic/right leg, while the target AGRF window was represented by gray vertical bars (Fig. 1b).^55^ Visual biofeedback exhibits proximity to the target and successful AGRF targeting (i.e., the X crossing into the target window). Auditory biofeedback was delivered as an audio tone played upon successfully achieving target AGRF during each gait cycle. Conventional biofeedback was delivered using The MotionMonitor software (Innovative Sport Training, Illinois, USA). Participants were provided verbal instructions that the “X” depicted the force exerted by their paretic/right foot pushing backward against the ground/treadmill. Participants were not explicitly instructed how to alter their push-off force.

##### Gamified biofeedback (gBF)

As with cBF, a screen and speaker were positioned in front of the participant for relaying visual and audio biofeedback. The custom game employs high-definition graphics compared to the simple display used by cBF. Audiovisual cues were provided through animated graphics in a game-like environment, centered around mining gems (Fig. 1c). An in-game pickaxe moved proportionally with the generation of paretic/right AGRF. Before beginning, participants were provided verbal instructions that the pickaxe movement in the game display represented the force exerted by their paretic/right foot against the ground, and were shown a 15 s video demonstration of how the game works. Similar to the cBF bout, participants were not instructed explicitly how to increase their push-off force. When participants successfully reached the target AGRF, the on-screen pickaxe visually strikes a rock, transforming it into a gem (visual biofeedback), accompanied by a twinkling celebratory sound (auditory biofeedback). Gems were then collected by a cart, rewarding participants with an incremental game score. A digital avatar, portrayed as a badger wearing a mining hat, was integrated into the game to relay encouraging statements. The gamified biofeedback was delivered using custom-written software designed by our study team (Unreal Engine, Epic Games, 2019, version 4.21.2).

## Data analysis

### Dependent variables

Gait biomechanics: Motion capture data were processed in Visual3D (C-Motion Inc., Maryland, USA). Primary dependent variables were peak anterior ground reaction force (AGRF) and trailing limb angle (TLA) of the right (AB) or paretic (ST) (targeted) leg. Targeted/paretic leg propulsion was measured as the peak anteriorly-directed ground reaction force during terminal stance. Trailing limb angle was calculated as the maximum posteriorly-directed angle between the laboratory’s vertical axis and a line joining the greater trochanter and fifth metatarsal head marker. Peak ankle moment was calculated as the peak ankle plantarflexion moment during stance phase. For each condition, gait biomechanics data were averaged across strides during walking trials, with the same number of strides used for analysis within each participant.

Physiological intensity: Heart rate was monitored throughout the session using a sensor placed on the chest under the clothing (Polar USA, Lake Success, NY), and data were captured at 1–2-minute intervals. To monitor self-report of intensity, participants were asked to report their perceived physical exertion using the Borg Scale of Perceived Exertion toward the end of each walking bout.^56^

Participant self-reports of engagement: To gauge user experience during biofeedback, participants completed three self-reports regarding the 2 biofeedback interfaces. The NASA Task Load Index (TLX) has been validated as a measure of subjective workload, and includes 6 subscales (mental demand, physical demand, temporal demand, performance, effort, frustration).^57^ The User Experience Questionnaire (UEQ), a reliable measure of user experience used to complement subjective quality ratings, was used to evaluate the 6 subscales of attractiveness, perspicuity, efficiency, dependability, stimulation, and novelty.^58^ Finally, 8-point Likert scales of boredom, motivation, enjoyment/fun, and challenge created by our lab were used to gain additional insight into participants’ subjective experiences during biofeedback.

### Statistical analysis

Statistical analyses for biomechanical and physiological intensity data were conducted using RStudio 2009–2021 version 2021.09.1 + 372.^59^ An adjusted p-value < 0.05 was considered significant and effect sizes of main and interaction effects were calculated using Epsilon-squared^60^. Our sample size was estimated based on the magnitude of effect size for biofeedback-induced change in peak AGRF in our previous published study on individuals post-stroke.^30^ Linear mixed effects models were used to determine the contributions of fixed effects of condition (3 conditions: noBF, cBF, gBF) on the % change in each variable from Pre to Min. 3 (immediate changes in gait with biofeedback on) and from Pre to Post (short-term changes in gait after biofeedback was switched off). Based on the lack of between-group (AB, ST) differences in the change in AGRF and TLA, the small sample size of the stroke cohort, and this being a preliminary study, analyses were pooled across the total sample (N = 15). To inform sample size estimation and design of future studies, we also conducted and reported the pairwise comparisons and effect sizes separately for each group (2 groups: able-bodied, stroke) (Supplementary Table 1). Null models created using the *lme* function in the *nlme* package were used to evaluate baseline differences in outcome measures due to random effects (Participant). Subsequent models incorporated fixed effects (condition) to determine significant main and interaction effects. ANOVAs were conducted using the *aov* function to calculate the likelihood ratio comparing the null model from those incorporating fixed effects. Pair-wise post hoc comparisons were calculated using the *emmeans* package with Bonferroni correction for multiple comparisons.

We used heart rate and rating of perceived exertion data from Min. 3 of each walking bout for our analyses. UEQ survey data were analyzed using the UEQ Data Analysis Tool, and Cohen’s d was calculated for each component scale.^61^ NASA-TLX survey scores were scaled to 100, representing perceived workload as defined in prior literature, then a two-tailed paired t-test was conducted to compare cBF versus gBF in Microsoft Excel (2016) for each component scale, similar to analysis for the UEQ.^57^ Likert survey data were similarly scaled to 100 and compared between cBF versus gBF using a two-paired paired t-test for each component scale. Because of the limited sample size in this feasibility study, both p-values and effect sizes were considered in discerning the impact of preliminary results.^62^ In addition to p-values < 0.05 indicating statistical significance, effect sizes were reported, with a Cohen’s d ≥ 0.5 being considered a medium effect size, and d ≥ 0.8 being considered a large effect size.^63^

## Results

All participants (AB and ST) completed the walking bouts. The participants understood and followed the instructions pertinent to each of the gait biofeedback interfaces (cBF and gBF). No discomfort, challenge, or adverse events were noted during the walking tests.

### Effects of cBF and gBF on gait biomechanics

#### Anterior ground reaction force (AGRF)

**Figure 2:**
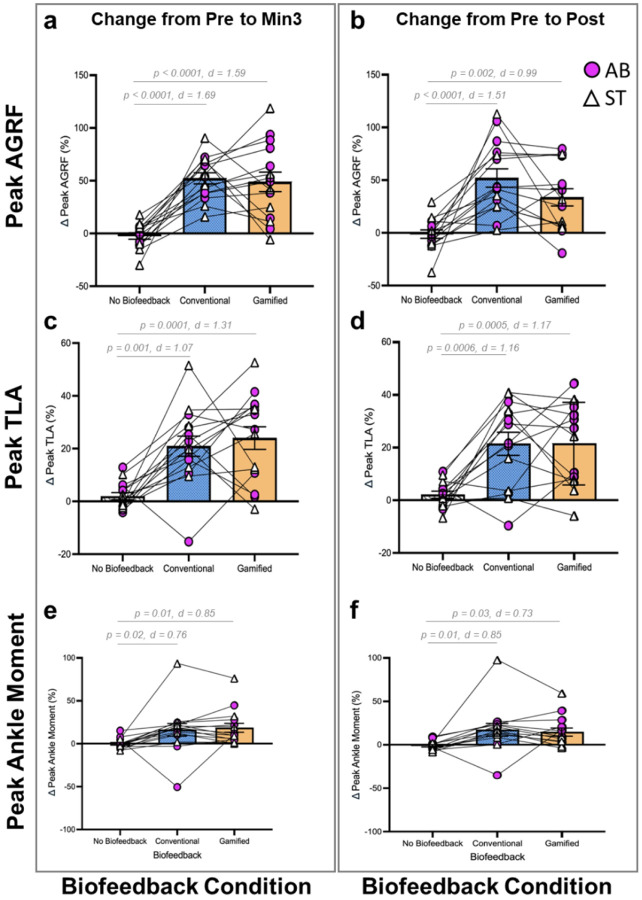
Effects of gait biofeedback conditions on gait biomechanics variables (peak anterior ground reaction force, trailing limb angle, and ankle moment). The graphs plot percent change from Pre to Min. 3 (left columns) and Pre to Post (right columns) for 3 gait biomechanics variables (peak AGRF (top row, a and b), TLA (middle row, c and d), and ankle moment (bottom row, e and f). Individual participant data for able-bodied (AB) and post-stroke (ST) participants are depicted. Bars indicate group means and standard error of the mean. For all pairwise comparisons of cBF and gBF with respect to the control condition (noBF), p-values and standardized effect sizes (d) are shown.

Analysis of percent change in AGRF from Pre to Min. 3 showed a significant main effect of condition (χ^2^(2) = 34.132, p < 0.0001, ^2^ = 0.59) (Fig. 2a). Compared to the no biofeedback (noBF) condition, there was a significantly larger percent change in AGRF during conventional biofeedback (cBF) (p < 0.0001, d = 1.69) and gamified biofeedback (gBF) (p < 0.0001, d = 1.59). There was no difference between cBF and gBF (p = 1.0, d = −0.10).

Analysis of percent change in AGRF from Pre to Post showed a significant main effect of condition (χ^2^(2) = 24.673, p < 0.0001, ^2^ = 0.51) (Fig. 2b). Compared to the noBF condition, there was a significantly larger percent change in AGRF for both the cBF (p < 0.0001, d = 1.51) and gBF conditions (p = 0.002, d = 0.99). There was no significant difference between cBF and gBF (p = 0.2, d = −0.52).

#### Trailing limb angle (TLA)

Analysis of percent change in TLA from Pre to Min. 3 showed a significant main effect of condition (χ^2^(2) = 20.935, p < 0.0001, ^2^ = 0.48) (Fig. 2c). Compared to the noBF condition, there was a significant increase in percent change in TLA during cBF (p = 0.001, d = 1.07) and gBF (p = 0.0001, d = 1.31). There was no difference between gBF and cBF (p = 1.0, d = 0.24).

Analysis of percent change in TLA from Pre to Post showed a significant main effect of condition (χ^2^(2) = 19.033, p = 0.0001, ^2^ = 0.46) (Fig. 2d). Compared to the noBF condition, there was a significant increase in percent change in TLA during cBF (p = 0.0006, d = 1.16) and gBF (p = 0.0005, d = 1.17). There was no significant difference between cBF and gBF (p = 1.0, d = 0.0).

#### Ankle moment

Analysis of percent change in ankle moment from Pre to Min. 3 showed a significant main effect of condition (χ^2^(2) = 10.857, p = 0.004, ^2^ = 0.31) (Fig. 2e). Compared to the noBF condition, there was a significant increase in percent change in ankle moment during cBF (p = 0.02, d = 0.76) and gBF (p = 0.01, d = 0.85). There was no significant difference between cBF and gBF (p = 1.0, d = 0.09).

Analysis of percent change in ankle moment from Pre to Post showed a main effect of condition (χ^2^(2) = 10.546, p = 0.005, ^2^ = 0.31) (Fig. 2f). Compared to noBF, pairwise comparisons showed a significantly greater percent change in ankle moment for cBF (p = 0.01, d = 0.85) and gBF (p = 0.03, d = 0.73). There was no significant difference between cBF and gBF (p = 1.0, d = −0.12).

#### Effects of cBF and gBF on physiological intensity Heart rate (HR)

**Figure 3:**
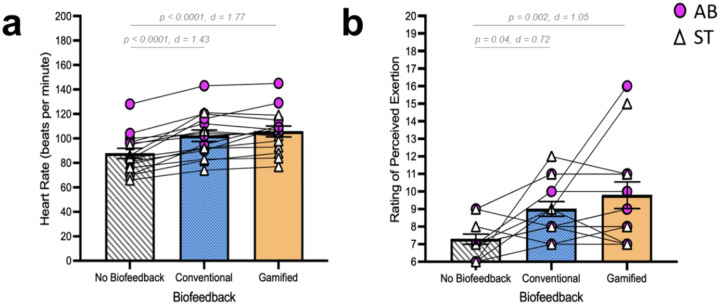
Effects of gait biofeedback conditions on physiological intensity (heart rate and rating of perceived exertion). Heart rate data collected during Min. 3 of the walking bouts (a) and RPE scores (b) are shown for the noBF, cBF, and gBF conditions. Individual participant data for able-bodied (AB) and post-stroke (ST) participants ae depicted. Bars indicate group means and standard error of the mean. For the pairwise comparisons of cBF and gBF with respect to the control condition (noBF), p-values and standardized effect sizes (d) are shown.

Analysis of HR data showed a significant main effect of condition (χ^2^(2) = 31.849, p < 0.0001, ^2^ = 0.57) (Fig. 3a). Compared to the noBF condition, there was a significant increase in HR during cBF (p < 0.0001, d = 1.43) and gBF (p < 0.0001, d = 1.77). There was no difference between cBF and gBF (p = 0.6, d = 0.34).

#### Rating of perceived exertion (RPE)

Analysis of RPE scores showed a significant main effect of condition (χ^2^(2) = 13.543, p = 0.001, ^2^ = 0.37) (Fig. 3b). Compared to the noBF condition, there was a significant increase in RPE during cBF (p = 0.04, d = 0.72) and gBF (p = 0.002, d = 1.05). There was no difference between cBF and gBF (p = 0.7, d = 0.33).

In addition to the above analyses, our pairwise comparisons conducted separately for the ST and AB groups showed medium (d ≥ 0.5) or large (d ≥ 0.8) effect sizes for cBF and gBF compared to noBF for the ST group for all gait biomechanics and physiological intensity variables (Supplementary Table 1).

**Supplementary Table 1.** Results for statistical comparisons of the effects of biofeedback (p-values, Cohen’s effect size (d)) for comparisons among noBF, cBF, and gBF gait conditions pooled (AB + ST) as well as separated separately for able-bodied (AB, n = 9) and stroke (ST, n = 6) participants. Moderate and large magnitude of effect sizes (Cohen’s d) are marked in blue and orange font, respectively.

#### Comparison of participant self-reports of engagement during cBF and gBF

##### NASA Task load index (TLX)

**Figure 4:**
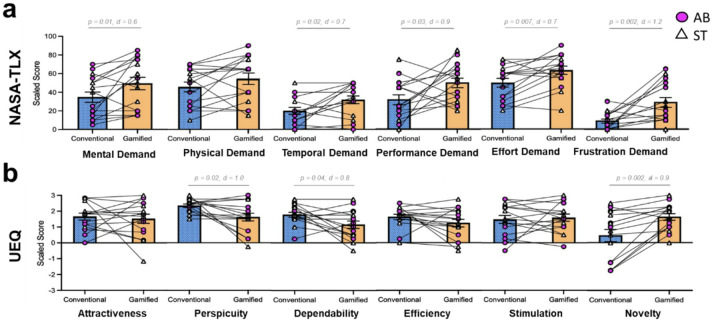
NASA-Task Load Index (NASA-TLX) and User Experience Questionnaire (UEQ) scores during the gait biofeedback conditions. (a) NASA-TLX self-reported scores are plotted for the cBF and gBF conditions for each of the 6 subscales: mental demand, physical demand, temporal demand, performance, effort, and frustration. (b) UEQ scores are plotted for the cBF and gBF conditions each of the component scales: attractiveness, perspicuity, dependability, efficiency, stimulation, novelty. (c) Likert scale. Only the challenge component scale score significantly differed between cBF and gBF, with participants rating the gBF interface as more challenging. Individual participant data for able-bodied (AB) and post-stroke (ST) participants ae depicted. Bars indicate group means and standard error of the mean. For the pairwise comparisons that were statistically significant (p < 0.05), both p-values and effect sizes (d) are indicated.

Five of the six component scales of the TLX showed significant differences between cBF and gBF (Fig. 4a). gBF was rated significantly higher than cBF for the mental demand (p = 0.01, d = 0.6), temporal demand (p = 0.02, d = 0.7), performance (p = 0.03, d = 0.9), effort (p = 0.007, d = 0.7), and frustration component scales (p = 0.002, d = 1.2). There was no significant difference in rating between the two biofeedback interfaces in the physical demand component scale (p = 0.1, d = 0.4).

##### User experience questionnaire (UEQ)

In the novelty component scale of the UEQ, gBF was rated significantly higher compared to cBF (p = 0.002, d = 0.9). In the perspicuity component scale, participants gave higher ratings to the cBF compared to gBF interface (p = 0.02, d = 1.0) (Fig. 4b). Similarly, the dependability rating was also significantly higher for cBF compared to gBF (p = 0.04, d = 0.8). The attractiveness (p = 0.7, d = 0.1), efficiency (p = 0.2, d = 0.5), and stimulation (p = 0.7, d = 0.1) component scales all showed no significant difference in user scoring between the two types of biofeedback interfaces.

##### Likert scale

Only the challenge component scale revealed significant differences between cBF and gBF, with gBF rating higher (p = 0.001, d = 1.0). The enjoyment/fun (p = 0.1, d = 0.6), boredom (p = 0.6, d = 0.1), and motivation (p = 0.8, d = 0.09) component scales showed high variability and did not significantly differ between cBF and gBF.

## Discussion

The current study showed that our novel, custom-designed gamified biofeedback interface for gait training was feasible, and we demonstrated its preliminary effects targeting propulsion during gait in able-bodied (AB) and stroke (ST) participants. Despite variability in participant ages and familiarity with gaming technology or biofeedback, all participants could safely complete the study conditions comprising conventional (cBF) and gamified biofeedback (gBF) without any adverse events. Compared to the control condition without biofeedback (noBF), both gBF and cBF showed significantly greater increases in the targeted (peak AGRF) and non-targeted (TLA, ankle moment) biomechanical variables during biofeedback exposure (Min. 3) and during the post-test without biofeedback exposure (Post), with moderate to large effect sizes. As expected, gBF and cBF were not significantly different from each other with regards to changes in AGRF or TLA. Compared to the no biofeedback walking condition, both biofeedback conditions also induced significant increases in HR and RPE, suggesting enhanced physiological intensity during the walking tasks with biofeedback. The NASA-TLX results showed that gBF induced a higher workload than cBF in the component scales of mental demand, temporal demand, performance, frustration, and effort. Also, participants’ scale self-reports rated gBF higher in the novelty component scale of the UEQ, and higher in the challenge component of the Likert scale. Taken together, our results show the feasibility of gamified gait biofeedback in able-bodied individuals and persons with a history of stroke, and we provide preliminary evidence of comparable short-term improvements in gait biomechanics and physiological intensity with both gamified and conventional biofeedback.

Previous work has demonstrated that conventional biofeedback induces increases in targeted gait biomechanical variables such as AGRF and TLA.^30, 40^ Here, we hypothesized that gamified gait biofeedback (gBF) would be feasible, and demonstrate similar increases in the targeted gait variables as conventional biofeedback (cBF). Overall, our findings show that our hypotheses were supported. We observed moderate to large effect sizes for both cBF and gBF versus the control (no BF) conditions, for gait biomechanics variables that were directly targeted by the biofeedback (AGRF) and related to AGRF (TLA, ankle moment). These results support the feasibility and promise of gBF as it elicited similar changes to biomechanical variables as cBF. Both cBF and gBF induced significant increases in AGRF for the targeted/paretic leg during biofeedback exposure. Despite the complexity of the gamified interface and aging- and stroke-related deficits, all participants were able to understand the audiovisual biofeedback interfaces and change their gait during biofeedback. As this study utilized a matched comparison of cBF and gBF at the same self-selected speed and AGRF targets for both interfaces, we expected—and observed—that both cBF and gBF would induce similar increases in gait biomechanics. We also compared physiological intensity using heart rate (HR) and rating of perceived exertion (RPE) during short bouts of walking with the novel gamified versus conventional biofeedback interfaces. Both AB and ST participants showed increases in HR and RPE during cBF and gBF compared to noBF, suggesting that gait biofeedback, whether conventional or gamified, can promote greater training intensity, which is consistent with recommendations from recent clinical practice guidelines for gait rehabilitation.^64^ Taken together, gamified gait biofeedback can enhance gait quality and promote greater intensity of practice, which can provide promising advantages as a gait retraining strategy.

Our initial hypothesis assumed that with similar effects on biomechanical variables, the engagement and fun provided by gamified gait biofeedback would be an added advantage compared to conventional biofeedback. We found mixed results pertaining to this hypothesis. We found significantly greater participant self-report scores on the NASA Task Load Index (TLX) for gBF versus cBF, with gBF inducing higher workload during walking compared to cBF for the subscales of mental demand, temporal demand, performance, effort, and frustration. gBF also scored 16% higher than cBF in physical demand (moderate effect size, d = 0.4), but this difference was not statistically significant (p = 0.14). Thus, our results suggest that gBF was considered more demanding by participants, which may be due to the greater novelty, complexity, and cognitive challenge of the gBF interface. We posit that the greater task demand imposed by gamified biofeedback provides an advantage, enhancing the challenge level and engagement during gait training. Potentially, greater exposure to gBF through additional training sessions may reduce the perceived task demands of gBF, when faster speeds or higher biofeedback targets would be needed to progress the training challenge. Fatigue may also contribute to the increased workload scores, whereby participants may need more time to adapt their gait, and will become more adjusted as training progresses. However, the lack of significant differences between gBF and cBF for the physical demand subscale of NASA-TLX appear to counteract fatigue as a contributing factor, and suggest that gBF may elicit a greater perception of cognitive workload instead of perceived physical demand.

To promote motor learning/relearning of gait patterns, an ideal motor training session should include repetitive, high intensity, task-specific, and challenging stepping practice, with high salience and engagement.^65–67^ In the unimpaired nervous system, task performance during locomotor control does not require overt attention to gait biomechanics or high cognitive effort, as subcortical circuits and central pattern generators enable automaticity of normal gait, with cortical centers being engaged only during visually-guided, complex, or attentional locomotor tasks.^68, 69^ However, in the presence of aging and neuropathologies such as stroke, neural control of gait may shift towards greater engagement of cortical centers.^70, 71^ Research studies on dynamic balance show variations in electroencephalographic-based measures of cortical engagement with task challenge and individual ability.^72–74^ Similarly, we predict that the increased workload and challenge ratings reported during gamified gait biofeedback may be accompanied by greater activation of motor and attentional cortical circuits compared to conventional biofeedback interfaces, which can be tested in future studies.^72–75^ Future work is also needed to optimize both the cognitive and motoric challenge points during gait training, as well as to identify neurophysiological and clinical biomarkers associated with response to gait biofeedback.

We also hypothesized that participants would report greater engagement and motivation, as well as lesser boredom and fatigue during gBF versus cBF. The UEQ results showed that novelty ratings were significantly higher for gBF compared to cBF, but gBF was rated lower for the perspicuity and dependability component scales. Contrary to our hypothesis, Likert results did not show greater motivation or lesser boredom with gBF versus cBF. Future design iterations of the gBF interface can further increase motivation, dependability, and fun, while maintaining higher levels of task demand. These designs should consider more customized games (e.g., aligned to an individual’s interest in Harry Potter or golf) with more responsive interfaces to help participants fully embody the information being provided by biofeedback, and translate the cues presented in a gamified interface into individual-specific, discrete biomechanical modifications. Tools like augmented reality that provide interactive, visualizationmerging digital displays with the user’s real-world environment may enhance the effectiveness of gamified biofeedback and enable overground training.^76^

Our current study aligns with previous research showing that the addition of video games to conventional rehabilitation interventions has the potential to enhance therapeutic benefits, enjoyment, and motivation. For example, the addition of a Nintendo Wii game to an already familiar task of treadmill walking or cycling increased exercise intensity (heart rate, cadence, speed).^49, 77^ Compared to balance platform therapy, video game therapy induced superior improvements in mobility and balance in people with traumatic brain injury.^78^ In frail, community-dwelling older adults, dynamic exercises on fixed and compliant surfaces were coupled to game-based exercise, resulting in improved balance.^79^ Off-the-shelf games such as Nintendo Wii and Microsoft Kinect have been used for therapy, and shown to increase enjoyment during rehabilitation in individuals with disabilities.^80–82^ With game use for upper limb rehabilitation, users stated that games made rehabilitation more fun and helped achieve greater exercise intensity.^80^ Games can provide opportunity to practice activities that may not be safe within the clinical environment.^44, 46, 47^ Persons engaged in rehabilitation deserve, and may, in due course, demand, high-quality, entertaining gaming interfaces during training. Regrettably, specialized games that target specific gait deficits are not yet available in rehabilitation clinics.^46, 47^ Thus, in light of the promising benefits of game-based interfaces for neurorehabilitation and the dearth of specific games for gait retraining, our current work is highly significant. By presenting the design, development, and preliminary testing of an innovative game-based biofeedback interface specifically custom-designed for gait retraining, we lay foundations for future research.

There are certain limitations inherent to testing different types of biofeedback interfaces. The gBF included visual cues of task performance including step count and score, as well as a timer showing how much time had elapsed. These cues were a necessary component of the game design, but due to these elements, gBF provided greater performance feedback to participants compared to cBF. Similarly, gBF provided verbal encouragement to participants during walking bouts (badger speaking encouraging phrases intermittently) whereas the cBF did not. Additionally, the cBF interface had an upper bound visual cue for target force, unlike gBF. This may contribute to participants becoming more efficient, with less excess force generated beyond the target AGRF level required. The gBF interface does not have this indicator, and participants may produce excessive force, leading to higher perceived task demands compared to cBF. Future biofeedback design iterations can explore different visualizations to indicate the biofeedback target threshold. Also, 2 ST participants with gait speeds < 0.5 m/s had to be excluded from the study because the gBF interface did not function at slower speeds, which limits applicability to persons with a history of stroke with severe gait deficits, and can be overcome in future iterations of game design. As an early design prototype, the gBF interface was often not as responsive as the cBF interface, creating a lag in the timing of audiovisual biofeedback. These occasional gBF design glitches may have contributed to lower ratings in the UEQ survey for gBF for dependability or perspicuity. Here, we did not evaluate longer durations of walking practice (e.g., 30 min) or longer-term recall tests to evaluate the persistence of altered biomechanical effects after removal of biofeedback. In future studies, gait training intensity can be increased by providing biofeedback at faster than self-selected walking speed as well as increasing the target AGRF % level, which may provide greater therapeutic benefits. One study provided a standardized method to select personalized biofeedback targets through prediction models based on speed, leg length, mass, sex, and age that can be implemented in future studies to mitigate the risk of over- or underestimating ideal target AGRF values.^83^ This work is represented as a small feasibility study, targeting the right leg in able-bodied individuals and persons with a history of stroke with right-sided hemiparesis. Larger sample sizes and the inclusion of more heterogenous cohorts of persons with a history of stroke will help elucidate the effects of gBF compared to cBF in future studies. Furthermore, there is a need to identify characteristics of responders and non-responders to gait biofeedback. Combining biofeedback with electroencephalography to measure cortical activity, or transcranial magnetic stimulation to measure cortical excitability, can provide insights into neural processes underlying gait biofeedback. Identifying neurophysiological, biomechanical, and clinical characteristics underlying response to gait biofeedback may inform more personalized and efficacious biofeedback approaches.

## Conclusions

We confirmed the feasibility of a novel, custom-designed gamified gait biofeedback interface targeting propulsion during gait, and demonstrated its similarity to a conventional biofeedback interface in inducing unilateral changes in targeted leg gait biomechanics and physiological intensity. Our results also showed that compared to conventional biofeedback, gamified biofeedback induced significantly greater mental, temporal, performance, effort, and frustration perceived task loads, as well as greater novelty and challenge. In summary, our results suggest that gBF is a feasible approach to target specific biomechanical variables while enhancing task demands during gait training, and warrants further investigation as a gait retraining intervention.

## Supplementary Files

This is a list of supplementary files associated with this preprint. Click to download.

- Onlinefloatimage4.png

## Figures and Tables

**Table 1:** Participant demographics.

Clinical Characteristics	Able-bodied (AB)	Stroke (ST)
Participants (n)	9	6
Age (years)	27.90 ± 4.1 0	63 ± 7.70
Sex (n male; n female)	4; 5	4; 2
Self-selected Gait Speed (m/s)	0.78 ± 0.19	0.58 ± 0.13
Target Anterior Ground Reaction Force (%)	21.70 ± 4.33	21.70 ± 4.08
Fugl-Meyer Lower Extremity Score (FMA-LE)	-	27.83 ± 2.48
Timed Up and Go Score (s)	-	8.35 ± 1.06

## Data Availability

The datasets used and/or analyzed during the current study are available from the corresponding author on reasonable request. The study results have also been shared on ClinicalTrials.gov at https://clinicaltrials.gov/study/NCT04013971 as part of the requirements for NIH-funded clinical trials.

## Abbreviations

AB: Able-bodied
AGRF: Anterior ground reaction force(s)
cBF: Conventional biofeedback
FMA-LE: Fugl-Meyer Assessment Lower Extremity
gBF: Gamified biofeedback
HR: Heart rate
NASA-TLX: National Aeronautics and Space Administration Task Load Index
noBF: No biofeedback/control
RPE: Rating of Perceived Exertion
ST: Stroke
TLA: Trailing limb angle
TUG: Timed Up and Go
UEQ: User Experience Questionnaire

## References

[1] Roger VL, Go AS, Lloyd-Jones DM, Benjamin EJ, Berry JD, Borden WB, Heart disease and stroke statistics–2012 update: a report from the American Heart Association. Circulation. 2012;125(1):e2–220. 22179539 10.1161/CIR.0b013e31823ac046 PMC4440543

[2] G. B. D. Stroke Collaborators. Global, regional, and national burden of stroke and its risk factors, 1990–2019: a systematic analysis for the Global Burden of Disease Study 2019. Lancet Neurol. 2021;20(10):795–820. 34487721 10.1016/S1474-4422(21)00252-0 PMC8443449

[3] Barbeau H, Visintin M. Optimal outcomes obtained with body-weight support combined with treadmill training in stroke subjects. Arch Phys Med Rehabil. 2003;84(10):1458–65. 14586912 10.1016/s0003-9993(03)00361-7

[4] Chen G, Patten C, Kothari DH, Zajac FE. Gait differences between individuals with post-stroke hemiparesis and non-disabled controls at matched speeds. Gait Posture. 2005;22(1):51–6. 15996592 10.1016/j.gaitpost.2004.06.009

[5] Granger CV, Hamilton BB, Gresham GE. The stroke rehabilitation outcome study–Part I: General description. Arch Phys Med Rehabil. 1988;69(7):506–9. 3389991

[6] Mayo NE, Wood-Dauphinee S, Ahmed S, Gordon C, Higgins J, McEwen S, Disablement following stroke. Disabil Rehabil. 1999;21(5–6):258–68. 10381238 10.1080/096382899297684

[7] Olney SJR. Hemiparetic gait following stroke. Part I: Characteristics. Gait Posture. 1996;4(2):136–48.

[8] Turnbull GI, Charteris J, Wall JC. A comparison of the range of walking speeds between normal and hemiplegic subjects. Scand J Rehabil Med. 1995;27(3):175–82. 8602480

[9] Teodoro J, Fernandes S, Castro C, Fernandes JB. Current Trends in Gait Rehabilitation for Stroke Survivors: A Scoping Review of Randomized Controlled Trials. J Clin Med. 2024;13(5). 10.3390/jcm13051358 PMC10932333 38592172

[10] Awad LN, Binder-Macleod SA, Pohlig RT, Reisman DS. Paretic Propulsion and Trailing Limb Angle Are Key Determinants of Long-Distance Walking Function After Stroke. Neurorehabil Neural Repair. 2015;29(6):499–508. 25385764 10.1177/1545968314554625 PMC4426250

[11] Balasubramanian CK, Bowden MG, Neptune RR, Kautz SA. Relationship between step length asymmetry and walking performance in subjects with chronic hemiparesis. Arch Phys Med Rehabil. 2007;88(1):43–9. 17207674 10.1016/j.apmr.2006.10.004

[12] Bowden MG, Balasubramanian CK, Behrman AL, Kautz SA. Validation of a speed-based classification system using quantitative measures of walking performance poststroke. Neurorehabil Neural Repair. 2008;22(6):672–5. 18971382 10.1177/1545968308318837 PMC2587153

[13] Bowden MG, Balasubramanian CK, Neptune RR, Kautz SA. Anterior-posterior ground reaction forces as a measure of paretic leg contribution in hemiparetic walking. Stroke. 2006;37(3):872–6. 16456121 10.1161/01.STR.0000204063.75779.8d

[14] Peterson CL, Hall AL, Kautz SA, Neptune RR. Pre-swing deficits in forward propulsion, swing initiation and power generation by individual muscles during hemiparetic walking. J Biomech. 2010;43(12):2348–55. 20466377 10.1016/j.jbiomech.2010.04.027 PMC2922425

[15] Awad LN, Reisman DS, Kesar TM, Binder-Macleod SA. Targeting paretic propulsion to improve poststroke walking function: a preliminary study. Arch Phys Med Rehabil. 2014;95(5):840–8. 24378803 10.1016/j.apmr.2013.12.012 PMC4160043

[16] Awad LN, Reisman DS, Pohlig RT, Binder-Macleod SA. Identifying candidates for targeted gait rehabilitation after stroke: better prediction through biomechanics-informed characterization. J Neuroeng Rehabil. 2016;13(1):84. 27663199 10.1186/s12984-016-0188-8 PMC5035477

[17] Bowden MG, Behrman AL, Neptune RR, Gregory CM, Kautz SA. Locomotor rehabilitation of individuals with chronic stroke: difference between responders and nonresponders. Arch Phys Med Rehabil. 2013;94(5):856–62. 23220082 10.1016/j.apmr.2012.11.032

[18] Hsiao H, Awad LN, Palmer JA, Higginson JS, Binder-Macleod SA. Contribution of Paretic and Nonparetic Limb Peak Propulsive Forces to Changes in Walking Speed in Individuals Poststroke. Neurorehabil Neural Repair. 2016;30(8):743–52. 26721869 10.1177/1545968315624780 PMC4930429

[19] Awad LN, Reisman DS, Pohlig RT, Binder-Macleod SA. Reducing The Cost of Transport and Increasing Walking Distance After Stroke: A Randomized Controlled Trial on Fast Locomotor Training Combined With Functional Electrical Stimulation. Neurorehabil Neural Repair. 2016;30(7):661–70. 26621366 10.1177/1545968315619696 PMC4885807

[20] Kim KH, Lee KB, Bae YH, Fong SSM, Lee SM. Effects of progressive backward body weight suppoted treadmill training on gait ability in chronic stroke patients: A randomized controlled trial. Technol Health Care. 2017;25(5):867–76. 28759977 10.3233/THC-160720

[21] Lin DJ, Finklestein SP, Cramer SC. New Directions in Treatments Targeting Stroke Recovery. Stroke. 2018;49(12):3107–14. 30571435 10.1161/STROKEAHA.118.021359 PMC6309806

[22] Saraiva J, Rosa G, Fernandes S, Fernandes JB. Current Trends in Balance Rehabilitation for Stroke Survivors: A Scoping Review of Experimental Studies. Int J Environ Res Public Health. 2023;20(19). 10.3390/ijerph20196829 PMC10572981 37835099

[23] Kleim JA, Jones TA. Principles of experience-dependent neural plasticity: implications for rehabilitation after brain damage. J Speech Lang Hear Res. 2008;51(1):S225–39. 18230848 10.1044/1092-4388(2008/018)

[24] Krakauer JW. Motor learning: its relevance to stroke recovery and neurorehabilitation. Curr Opin Neurol. 2006;19(1):84–90. 16415682 10.1097/01.wco.0000200544.29915.cc

[25] Krakauer JW, Carmichael ST, Corbett D, Wittenberg GF. Getting neurorehabilitation right: what can be learned from animal models? Neurorehabil Neural Repair. 2012;26(8):923–31. 22466792 10.1177/1545968312440745 PMC4554531

[26] Lang CE, Macdonald JR, Reisman DS, Boyd L, Jacobson Kimberley T, Schindler-Ivens SM, Observation of amounts of movement practice provided during stroke rehabilitation. Arch Phys Med Rehabil. 2009;90(10):1692–8. 19801058 10.1016/j.apmr.2009.04.005 PMC3008558

[27] Bowden MG, Woodbury ML, Duncan PW. Promoting neuroplasticity and recovery after stroke: future directions for rehabilitation clinical trials. Curr Opin Neurol. 2013;26(1):37–42. 23254556 10.1097/WCO.0b013e32835c5ba0

[28] Spencer J, Wolf SL, Kesar TM. Biofeedback for Post-stroke Gait Retraining: A Review of Current Evidence and Future Research Directions in the Context of Emerging Technologies. Front Neurol. 2021;12:637199. 33859607 10.3389/fneur.2021.637199 PMC8042129

[29] Finley JM, Gotsis M, Lympouridis V, Jain S, Kim A, Fisher BE. Design and Development of a Virtual Reality-Based Mobility Training Game for People With Parkinson’s Disease. Front Neurol. 2020;11:577713. 33519665 10.3389/fneur.2020.577713 PMC7843522

[30] Genthe K, Schenck C, Eicholtz S, Zajac-Cox L, Wolf S, Kesar TM. Effects of real-time gait biofeedback on paretic propulsion and gait biomechanics in individuals post-stroke. Top Stroke Rehabil. 2018;25(3):186–93. 29457532 10.1080/10749357.2018.1436384 PMC5901660

[31] Giggins OM, Persson UM, Caulfield B. Biofeedback in rehabilitation. J Neuroeng Rehabil. 2013;10:60. 23777436 10.1186/1743-0003-10-60 PMC3687555

[32] Druzbicki M, Guzik A, Przysada G, Kwolek A, Brzozowska-Magon A. Efficacy of gait training using a treadmill with and without visual biofeedback in patients after stroke: A randomized study. J Rehabil Med. 2015;47(5):419–25. 25757954 10.2340/16501977-1949

[33] Jonsdottir J, Cattaneo D, Recalcati M, Regola A, Rabuffetti M, Ferrarin M, Task-oriented biofeedback to improve gait in individuals with chronic stroke: motor learning approach. Neurorehabil Neural Repair. 2010;24(5):478–85. 20053951 10.1177/1545968309355986

[34] Jonsdottir J, Cattaneo D, Regola A, Crippa A, Recalcati M, Rabuffetti M, Concepts of motor learning applied to a rehabilitation protocol using biofeedback to improve gait in a chronic stroke patient: an A-B system study with multiple gait analyses. Neurorehabil Neural Repair. 2007;21(2):190–4. 17312094 10.1177/1545968306290823

[35] Lewek MD, Feasel J, Wentz E, Brooks FP Jr., Whitton MC. Use of visual and proprioceptive feedback to improve gait speed and spatiotemporal symmetry following chronic stroke: a case series. Phys Ther. 2012;92(5):748–56. 22228605 10.2522/ptj.20110206 PMC3345339

[36] Wolf SL. Electromyographic biofeedback applications to stroke patients. A critical review. Phys Ther. 1983;63(9):1448–59. 6351119 10.1093/ptj/63.9.1448

[37] Wolf SL, Binder-MacLeod SA. Electromyographic biofeedback applications to the hemiplegic patient. Changes in lower extremity neuromuscular and functional status. Phys Ther. 1983;63(9):1404–13. 6611661 10.1093/ptj/63.9.1404

[38] Franz JR, Maletis M, Kram R. Real-time feedback enhances forward propulsion during walking in old adults. Clin Biomech (Bristol). 2014;29(1):68–74. 24238977 10.1016/j.clinbiomech.2013.10.018

[39] Liu J, Santucci V, Eicholtz S, Kesar TM. Comparison of the effects of real-time propulsive force versus limb angle gait biofeedback on gait biomechanics. Gait Posture. 2021;83:107–13. 33129170 10.1016/j.gaitpost.2020.10.014 PMC7787119

[40] Schenck C, Kesar TM. Effects of unilateral real-time biofeedback on propulsive forces during gait. J Neuroeng Rehabil. 2017;14(1):52. 28583196 10.1186/s12984-017-0252-z PMC5460355

[41] Barr PN, Biddle J. Video game values: Human–computer interaction and games. Interact Comput. 2006;19(2):180–95.

[42] Lockton D, Harrison D, Stanton NA. The Design with Intent Method: a design tool for influencing user behaviour. Appl Ergon. 2010;41(3):382–92. 19822311 10.1016/j.apergo.2009.09.001

[43] Malone TW. What makes things fun to learn? heuristics for designing instructional computer games. Proceedings of the 3rd ACM SIGSMALL Symposium and the First SIGPC Symposium on Small Systems. {New York, NY, USA}: Association for Computing Machinery; 1980. pp. 162–9, numpages = 8.

[44] Henderson A, Korner-Bitensky N, Levin M. Virtual reality in stroke rehabilitation: a systematic review of its effectiveness for upper limb motor recovery. Top Stroke Rehabil. 2007;14(2):52–61. 10.1310/tsr1402-52 17517575

[45] Keshner EA. Virtual reality and physical rehabilitation: a new toy or a new research and rehabilitation tool? J Neuroeng Rehabil. 2004;1(1):8. 15679943 10.1186/1743-0003-1-8 PMC546404

[46] Laver KE, George S, Thomas S, Deutsch JE, Crotty M. Virtual reality for stroke rehabilitation. Cochrane Database Syst Rev. 2015;2015(2):CD008349. 10.1002/14651858.CD008349.pub3 PMC6465102 25927099

[47] Lohse KR, Hilderman CG, Cheung KL, Tatla S, Van der Loos HF. Virtual reality therapy for adults post-stroke: a systematic review and meta-analysis exploring virtual environments and commercial games in therapy. PLoS ONE. 2014;9(3):e93318. 24681826 10.1371/journal.pone.0093318 PMC3969329

[48] Weiss PL, Rand D, Katz N, Kizony R. Video capture virtual reality as a flexible and effective rehabilitation tool. J Neuroeng Rehabil. 2004;1(1):12. 15679949 10.1186/1743-0003-1-12 PMC546410

[49] Gallagher R, Werner WG, Damodaran H, Deutsch JE. Influence of cueing, feedback and directed attention on cycling in a virtual environment: Preliminary findings in healthy adults and persons with Parkinson’s disease. 2015. pp. 11–7.

[50] Levac DE, Glegg SM, Sveistrup H, Colquhoun H, Miller P, Finestone H, Promoting Therapists’ Use of Motor Learning Strategies within Virtual Reality-Based Stroke Rehabilitation. PLoS ONE. 2016;11(12):e0168311. 27992492 10.1371/journal.pone.0168311 PMC5167266

[51] Sveistrup H. Motor rehabilitation using virtual reality. J Neuroeng Rehabil. 2004;1(1):10. 15679945 10.1186/1743-0003-1-10 PMC546406

[52] Lawson RR, Gayle JO, Wheaton LA. Novel behavioral indicator of explicit awareness reveals temporal course of frontoparietal neural network facilitation during motor learning. PLoS ONE. 2017;12(4):e0175176. 28410404 10.1371/journal.pone.0175176 PMC5391991

[53] Taylor JA, Ivry RB. Implicit and Explicit Processes in Motor Learning. In: Prinz W, Beisert M, Herwig A, editors. Action Science: Foundations of an Emerging Discipline. The MIT Press; 2013. p. 0.

[54] Kesar TM, Binder-Macleod SA, Hicks GE, Reisman DS. Minimal detectable change for gait variables collected during treadmill walking in individuals post-stroke. Gait Posture. 2011;33(2):314–7. 21183350 10.1016/j.gaitpost.2010.11.024 PMC3042506

[55] Liu J, Kim HB, Wolf SL, Kesar TM. Comparison of the Immediate Effects of Audio, Visual, or Audiovisual Gait Biofeedback on Propulsive Force Generation in Able-Bodied and Post-stroke Individuals. Appl Psychophysiol Biofeedback. 2020;45(3):211–20. 32347399 10.1007/s10484-020-09464-1 PMC7447533

[56] Borg G. Perceived exertion as an indicator of somatic stress. Scand J Rehabil Med. 1970;2(2):92–8. 5523831

[57] Hart SGS. Development of NASA-TLX (Task Load Index): Results of Empirical and Theoretical Research. In: Peter AH, Najmedin M, editors. Human Mental Workload. Volume 52. North-Holland; 1988. pp. 139–83.

[58] Laugwitz BS, Held M. Konstruktion eines Fragebogens zur Messung der User Experience von Softwareprodukten. In: Heinecke HM, Paul H, editors. Mensch und Computer 2006. München: Oldenbourg Wissenschafts; 2006. pp. 125–34.

[59] R Core Team. A language and environment for statistical computing. Version 4.1.2. 4.1.2 ed. R Foundation for Statistical Computing; 2017.

[60] Mangiafico SS. Summary and analysis of extension program evaluation in R, version 1.19.10. New Brunswick, NJ: Rutgers Cooperative Extension; 2016.

[61] Schrepp MH, Thomaschewski A. J. User Experience Questionnaire Data Analysis Tools. 2024.

[62] Lakens D. Calculating and reporting effect sizes to facilitate cumulative science: a practical primer for t-tests and ANOVAs. Front Psychol. 2013;4:863. 24324449 10.3389/fpsyg.2013.00863 PMC3840331

[63] Cohen J. Statistical Power Analysis for the Behavioral Sciences. 2nd ed. New York: Routledge; 1988.

[64] Hornby TG, Reisman DS, Ward IG, Scheets PL, Miller A, Haddad D, Clinical Practice Guideline to Improve Locomotor Function Following Chronic Stroke, Incomplete Spinal Cord Injury, and Brain Injury. J Neurol Phys Ther. 2020;44(1):49–100. 31834165 10.1097/NPT.0000000000000303

[65] Cramer SC, Sur M, Dobkin BH, O’Brien C, Sanger TD, Trojanowski JQ, Harnessing neuroplasticity for clinical applications. Brain. 2011;134(Pt 6):1591–609. 21482550 10.1093/brain/awr039 PMC3102236

[66] Hornby TG, Straube DS, Kinnaird CR, Holleran CL, Echauz AJ, Rodriguez KS, Importance of specificity, amount, and intensity of locomotor training to improve ambulatory function in patients poststroke. Top Stroke Rehabil. 2011;18(4):293–307. 21914594 10.1310/tsr1804-293

[67] Wolpaw JR. Harnessing neuroplasticity for clinical applications. Brain. 2012;135(Pt 4):e215. author reply e6. 22374936 10.1093/brain/aws017 PMC3326250

[68] Patla AE. Understanding the roles of vision in the control of human locomotion. Gait Posture. 1997;5(1):54–69.

[69] Ramadan R, Geyer H, Jeka J, Schoner G, Reimann H. A neuromuscular model of human locomotion combines spinal reflex circuits with voluntary movements. Sci Rep. 2022;12(1):8189. 35581211 10.1038/s41598-022-11102-1 PMC9114145

[70] Jahn K, Deutschländer AB, Stephan T, Kalla R, Hüfner K, Wagner J, Supraspinal locomotor control in quadrupeds and humans. Prog Brain Res. 2008;171:353–62. 18718326 10.1016/S0079-6123(08)00652-3

[71] Takakusaki K. Neurophysiology of gait: from the spinal cord to the frontal lobe. Mov Disord. 2013;28(11):1483–91. 24132836 10.1002/mds.25669

[72] Boebinger SE, Payne AM, Xiao J, Martino G, Borich M, McKay JL Cortically-mediated muscle responses to balance perturbations increase with perturbation magnitude in older adults with and without Parkinson’s disease. bioRxiv. 2024:2024.12.09.627582. 10.1523/ENEURO.0423-25.2026 PMC13064419 41871956

[73] Mirdamadi JL, Ting LH, Borich MR. Distinct Cortical Correlates of Perception and Motor Function in Balance Control. J Neurosci. 2024;44(15). 10.1523/JNEUROSCI.1520-23.2024 PMC11007305 38413231

[74] Palmer JA, Payne AM, Mirdamadi JL, Ting LH, Borich MR. Delayed Cortical Responses During Reactive Balance After Stroke Associated With Slower Kinetics and Clinical Balance Dysfunction. Neurorehabil Neural Repair. 2025;39(1):16–30. 39328051 10.1177/15459683241282786 PMC11723813

[75] Boebinger S, Payne A, Martino G, Kerr K, Mirdamadi J, McKay JL, Precise cortical contributions to sensorimotor feedback control during reactive balance. PLoS Comput Biol. 2024;20(4):e1011562. 38630803 10.1371/journal.pcbi.1011562 PMC11057980

[76] Riva G, Banos RM, Botella C, Mantovani F, Gaggioli A. Transforming Experience: The Potential of Augmented Reality and Virtual Reality for Enhancing Personal and Clinical Change. Front Psychiatry. 2016;7:164. 27746747 10.3389/fpsyt.2016.00164 PMC5043228

[77] Deutsch JE, Rothman J, Barker B, Grando A, Damodaran H. The effect of video game interaction on walking intensity: Preliminary study of young, older adults and persons post-stroke. 2015. pp. 24–9.

[78] Straudi S, Severini G, Sabbagh Charabati A, Pavarelli C, Gamberini G, Scotti A, The effects of video game therapy on balance and attention in chronic ambulatory traumatic brain injury: an exploratory study. BMC Neurol. 2017;17(1):86. 28490322 10.1186/s12883-017-0871-9 PMC5424286

[79] Szturm T, Betker AL, Moussavi Z, Desai A, Goodman V. Effects of an interactive computer game exercise regimen on balance impairment in frail community-dwelling older adults: a randomized controlled trial. Phys Ther. 2011;91(10):1449–62. 21799138 10.2522/ptj.20090205

[80] Lange B, Chang CY, Suma E, Newman B, Rizzo AS, Bolas M. Development and evaluation of low cost game-based balance rehabilitation tool using the Microsoft Kinect sensor. Annu Int Conf IEEE Eng Med Biol Soc. 2011;2011:1831–4. 22254685 10.1109/IEMBS.2011.6090521

[81] Saposnik G, Teasell R, Mamdani M, Hall J, McIlroy W, Cheung D, Effectiveness of virtual reality using Wii gaming technology in stroke rehabilitation: a pilot randomized clinical trial and proof of principle. Stroke. 2010;41(7):1477–84. 20508185 10.1161/STROKEAHA.110.584979 PMC4879973

[82] Yong Joo L, Soon Yin T, Xu D, Thia E, Pei Fen C, Kuah CW, A feasibility study using interactive commercial off-the-shelf computer gaming in upper limb rehabilitation in patients after stroke. J Rehabil Med. 2010;42(5):437–41. 20544153 10.2340/16501977-0528

[83] Bonilla Yanez MK, Finley SA, Schweighofer JM, Leech N. Gait speed and individual characteristics are related to specific gait metrics in neurotypical adults. Sci Rep. 2023;13(1):8069. 37202435 10.1038/s41598-023-35317-y PMC10195830

